# Microbe-dependent lymphatic migration of neutrophils modulates lymphocyte proliferation in lymph nodes

**DOI:** 10.1038/ncomms8139

**Published:** 2015-05-14

**Authors:** Henry R. Hampton, Jacqueline Bailey, Michio Tomura, Robert Brink, Tatyana Chtanova

**Affiliations:** 1 Immunology Division, The Garvan Institute of Medical Research, 384 Victoria street, Darlinghurst, New South Wales 2010, Australia; 2 St Vincent's Clinical School, Faculty of Medicine, UNSW Australia, 390 Victoria street, Darlinghurst, New South Wales 2010, Australia; 3 Laboratory of Immunology Faculty of Pharmacy, Osaka-Ohtani University 3-11-1 Nishikiorikita,Tondabayashi-city, Osaka prefecture, 584-8540, Japan

## Abstract

Neutrophil recruitment to the site of injury is an essential first step of an anti-bacterial response. However, little is known about the basis for and relevance of neutrophil migration from inflamed tissue into lymphoid organs. We established a photoconversion-based system to monitor the fate of neutrophils recruited to inflamed skin. While neutrophils are efficiently recruited to sites of both microbial and sterile lesions, subsequent re-localization to draining lymph nodes happens only when bacteria are present in the primary lesion. Skin egress of neutrophils occurs via lymphatic vessels and is dependent on CD11b and CXCR4 but not CCR7. Neutrophils are the predominant immune cell to migrate from inflamed skin into lymph nodes where they augment lymphocyte proliferation. Furthermore, inhibition of neutrophil migration from skin reduces T-cell proliferation in draining lymph nodes. Thus neutrophils mediate rapid cellular communication between the initial injury site and secondary lymphoid organs and modulate immune responsiveness.

## Abstract (web summary)

Both sterile and microbial injuries lead to rapid neutrophil recruitment to the site of inflammation. Here the authors show that only neutrophils responding to microbial ligands leave inflamed skin and migrate to draining lymph nodes where they stimulate lymphocyte proliferation.

## Graphical abstract

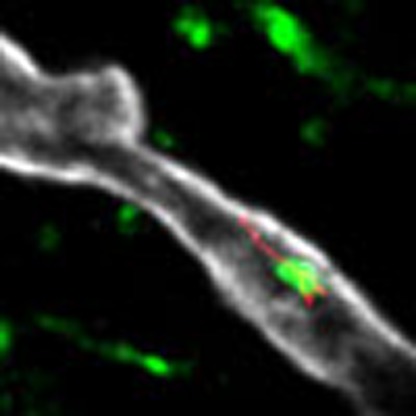

Neutrophils are the first leukocytes recruited to sites of infection or tissue damage, where they act by killing invading bacteria and promoting wound repair^1^,^2^,^3^. These properties of neutrophils have been recognized for several decades, the prevailing view being that they perform these functions following recruitment into inflamed tissue before dying *in situ.* The recent discovery that neutrophils in inflamed tissues exhibit a greatly extended lifespan of up to 5 days^4^ suggests that these cells may participate in more complex activities such as shaping of innate and adaptive immune responses. Moreover, observations in humans^5^, mice^6^ and zebrafish^7^ suggest that neutrophils can re-enter the vasculature from inflamed tissues via reverse transmigration. The possibility that neutrophils may also emigrate from inflamed tissues via the lymphatics has also been supported by several studies analysing neutrophil localization in the lymph nodes in mice^8^,^9^,^10^ as well as by analysis of afferent lymph in sheep^11^,^12^,^13^.

The potential for neutrophils to both survive for an extended period of time and migrate from sites of primary inflammation has led to a re-assessment of the role of these cells beyond direct pathogen destruction and wound repair. Neutrophils produce an array of molecules that recruit and activate other innate cells including monocytes, macrophages and dendritic cells^2^. In addition, a possible contribution by neutrophils in modulating adaptive immune responses has been supported by studies demonstrating that they can influence CD8^+^ T-cell responses by cross-presenting exogenous antigens *in vivo*^14^. Other studies suggest that neutrophils may present antigen to CD4^+^ T cells either directly^15^,^16^ or by fusing with DCs to form a hybrid cell population^17^,^18^. However, the net effect of neutrophils on T-cell responses is likely to be contributed to by multiple neutrophil functions including removal and sequestration of antigen^10^, modulation of APC maturation and function^10^, inhibition of T-cell migration^19^ as well as direct antigen presentation^15^,^16^. Several recent studies have also highlighted a possible role for neutrophils in regulating B-cell responses^20^,^21^.

Despite accumulating evidence that neutrophils can migrate out of primary inflammatory sites and potentially modify innate and adaptive immune responses, many questions remain unanswered. The differences in neutrophil behaviour in response to sterile and microbial inflammation remain to be characterized. In addition, the molecular basis for neutrophil migration from the site of primary injury to local lymph nodes has not been elucidated.

Here we describe a novel, photoconversion-based system designed to label cells that have migrated into inflamed skin and track them as they egress from a site of primary inflammation. While neutrophils entered inflamed skin in response to both bacterial and sterile injuries, migration to draining lymph nodes was dependent on the presence of bacteria. Neutrophils were the predominant leukocyte to leave microbial lesions and migrate via the lymphatic vessels to the draining lymph node in a process dependent on CD11b and CXCR4 but not CCR7. Although neutrophil persistence in the lymph nodes was reduced in comparison to inflamed skin, we found that skin-egressing neutrophils were able to augment lymphocyte proliferation in draining lymph nodes. These data indicate a previously unappreciated level of communication between pathogens and neutrophils that promotes lymphatic migration of neutrophils and modulates downstream immune responses.

## Results

### Neutrophil recruitment to microbial and sterile lesions

**Figure 1:**
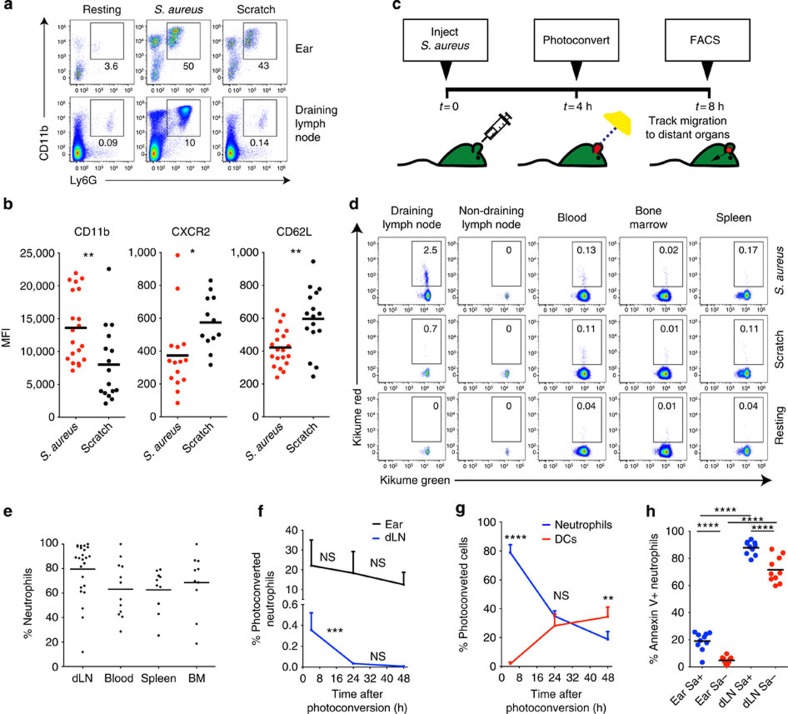
Neutrophils migrate from skin following microbial but not sterile inflammation. (**a**) Flow cytometric analysis of mouse ears and draining lymph nodes 8 h after an injection of killed *S. aureus* (middle panel) or a scratch with a sterile needle (right panel). Percentages of neutrophils (Ly6G^+^CD11b^+^) out of all live cells are shown. Resting controls: left panel. Fluorescence-activated cell sorting (FACS) profiles are representative of greater than four independent experiments with at least two mice per group. (**b**) Cell surface expression of the indicated molecules on neutrophils recruited to skin following an injection of *S. aureus* or needle scratch was analysed by flow cytometry 8 h after challenge. Each circle represents a single ear. (**c**) Experimental setup for the detection of skin-emigrant cells. Ears of Kikume reporter mice were either infected with *S. aureus* or scratched with a sterile needle, and, 4 h later, ears were photoconverted by exposure to white light for 20 min. A further 4 h later, mice were killed and photoconverted cells were detected by flow cytometry. (**d**) Flow cytometric detection of photoconverted neutrophils after mice were treated as described in **c**. FACS profiles are representative of at least six independent experiments and are gated on total neutrophils. (**e**) Proportion of neutrophils out of all photoconverted cells found in each organ using flow cytometry. Kikume mice were injected with *S. aureus*, photoconverted and analysed as described in **c**. Each circle represents a single organ. Data were pooled from four independent experiments with at least three mice per group. (**f**) Percentage of photoconverted neutrophils out of all live cells in ears and draining lymph nodes over time. Mice were infected with *S. aureus* as in **c**, photoconverted 4 h later. Means+s.d. are shown. (**g**) Identity of red cells in draining lymph nodes at 4, 24 and 48 h after photoconversion. DCs, dendritic cells. Mice were infected as in **c**. Means+s.d. are shown. (**h**) Percentage of Annexin V+ neutrophils in ears and draining lymph nodes 8 h after injection of fluorescently labelled *S. aureus*. Each circle represents a single organ. *S. aureus* (Sa)^+^ neutrophils (blue), *S. aureus*^−^ neutrophils (red). Data were analysed using an unpaired student's *t*-test (**b**), one-way analysis of variance (ANOVA) with a Bonferroni correction for multiple comparisons (**f**) or a two-way ANOVA with a Bonferroni correction (**g**,**h**). *P≤0.05; **P≤0.01; ***P≤0.001; ****P≤0.0001; NS, not significant.

To compare phenotypes and behaviour of neutrophils during the early stages of anti-bacterial and sterile immune responses, fluorescently conjugated and killed *S. aureus* was introduced intradermally into the mouse ear. A second cohort of mice received a sterile injury to the ear skin via a needle scratch while control mice remained unmanipulated. After 8 h, both sterile and bacterial injuries induced the recruitment of neutrophils (defined as Ly6G and CD11b positive) into the inflamed skin (Fig. 1a top panel and Supplementary Fig. 1). However, accumulation of neutrophils in dorsal cervical lymph nodes that drain the ear was only observed when *S. aureus* bacteria were present in the primary lesion (Fig. 1a bottom panel and Supplementary Fig. 1). Flow cytometric analysis of neutrophils recruited to the skin in response to microbial or sterile injuries revealed differential expression of molecules associated with neutrophil migration and activation. Specifically, CD11b was preferentially expressed on neutrophils recruited in response to *S. aureus*, while CD62L and CXCR2 were higher on neutrophils responding to sterile injury (Fig. 1b). Thus, despite similar levels of neutrophil recruitment to sites of bacterial and sterile inflammation, neutrophils show phenotypic and migration differences very early in the response.

### Neutrophils emigrate from microbial but not sterile lesions

To determine whether the neutrophils initially recruited to the primary skin wound underwent subsequent migration to other organs or remained in the skin, we utilized transgenic mice that ubiquitously express the coral-derived fluorescent protein Kikume. Kikume has a native green fluorescence but can be irreversibly converted to red fluorescence by exposure to ultraviolet or violet light^22^. The experimental approach used is summarized in Fig. 1c. First, Kikume transgenic mice were challenged by ear injection of *S. aureus* or a sterile needle scratch. After 4 h (a time point chosen to allow for neutrophil recruitment into skin), inflamed ears were photoconverted by exposure to white light as previously described^23^. A further 4 h later, mice were killed and photoconverted cells were detected in blood, draining lymph nodes and other immune organs using flow cytometry. In mice challenged with *S. aureus*, substantial numbers of photoconverted neutrophils were found in draining but not in non-draining lymph nodes (Fig. 1d top panel and Supplementary Fig. 2a). Photoconverted neutrophils could also be detected in peripheral blood of these mice but at 10-fold lower frequencies compared with draining lymph nodes (Fig. 1d and Supplementary Fig. 2b). In contrast, mice receiving a sterile scratch to the ear showed few photoconverted neutrophils in the draining lymph nodes despite the presence of these cells in the blood (Fig. 1d middle panel and Supplementary Fig. 2b). Flow cytometric analysis revealed that neutrophils comprised ∼80% of the photoconverted cells found in the draining lymph nodes of *S. aureus*-injected mice as well as the majority of the photoconverted cells in all other organs (Fig. 1e).

Although neutrophils recruited in response to *S. aureus* persisted in the ear for several days, the number of photoconverted neutrophils in draining lymph nodes rapidly declined and returned to basal levels ∼36 h after infection (Fig. 1f). By 48 h after challenge, dendritic cells were the predominant skin-emigrant population in draining lymph nodes (Fig. 1g). This decline in neutrophil numbers is likely due to both a decrease in migration as well as rapid death of lymph node neutrophils as suggested by substantially higher staining for the apoptotic marker Annexin V in draining lymph node neutrophils compared with those in skin (Fig. 1h). Annexin V binding was increased in both *S. aureus*-containing and non-containing draining lymph node neutrophils (Fig. 1h). Thus, neutrophils were the major immune subset to emigrate from inflamed skin at the very early stages of the immune response and their migration to draining lymph nodes was dependent on the presence of bacteria.

### Neutrophils migrate from skin to lymph nodes via lymphatics

**Figure 2:**
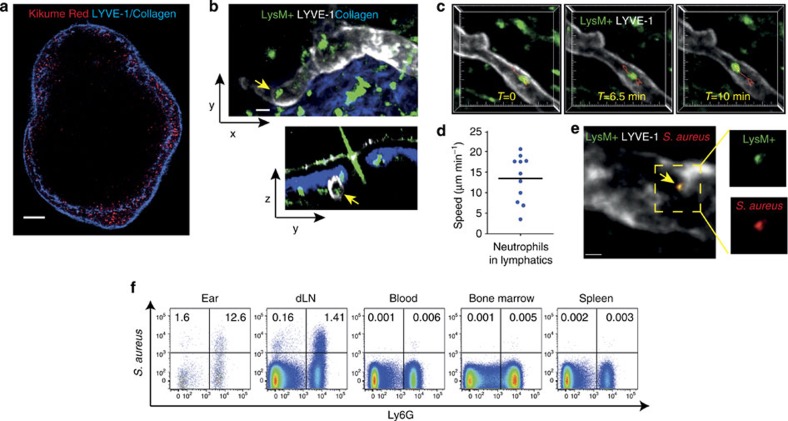
Neutrophils migrate from skin to lymph nodes via lymphatic vessels. (**a**) Kikume reporter mice were injected with *S. aureus* and photoconverted 4 h later. Draining lymph nodes were excised after 2–4 h and photoconverted cells (red) were visualized using two-photon microscopy. A single optical section of the entire lymph node is shown. Anti-LYVE-1 (blue) was injected into the ear to label the lymphatic vessels. Scale bar represents 100 μm. (**b**) Two-photon image of Lysozyme M GFP (LysM) reporter mouse ear 6 h after *S. aureus* injection. Arrows point to Lysozyme M^+^ (green) neutrophils inside the LYVE-1^+^ lymphatic vessels (white), collagen (blue). xy and yz planes for the same imaging volume are shown. Scale bar represents 20 μm. (**c**) A maximum intensity projection of a three-dimensional volume acquired via two-photon microscopy. Lysozyme M^+^ GFP neutrophil (green) migrating inside a lymphatic vessel (LYVE-1, white). Three representative time points are shown. Red track indicates neutrophil's path. Tick marks are 10 μm apart. (**d**) Lysozyme M GFP bone marrow was transferred into albino.BL6 mice 24 h before imaging. Ear skin was imaged in live mice 2–6 h following injection of *S. aureus*. The speed of neutrophils inside LYVE-1^+^ (white) lymphatic vessels was quantified. (**e**) Two-photon image of Lysozyme M GFP reporter mouse ear 6 h after fluorescent *S. aureus* injection. Arrow points to a Lysozyme M+ (green) neutrophil containing *S. aureus* (red) inside the LYVE-1^+^ lymphatics (white). Outlined area (left) corresponds to enlargement (right). Scale bar represents 10 μm. (**f**) Flow cytometric analysis of *S. aureus*-containing cells in different organs, 8 h after injection of fluorescent *S. aureus*. dLN, draining lymph node. Fluorescence-activated cell sorting profiles are representative of seven independent experiments with at least two mice per group and are gated on all live cells.

From the data presented in Fig. 1d, it was clear that migration of skin-egressing neutrophils to draining lymph nodes required the presence of *S. aureus* whereas reverse transmigration into blood vessels did not. We, therefore, postulated that the afferent lymphatic vessels rather than blood were the major route of neutrophil egress from the bacterial skin lesion to the draining lymph node. To distinguish between blood versus lymphatic migration of neutrophils from the skin wound to draining lymph nodes, we first examined the localization of photoconverted cells within the draining nodes of Kikume transgenic mice. Two-photon imaging after *S. aureus* injection and ear photoconversion showed that 4–6 h after infection, the majority of photoconverted cells were located in the subcapsular sinus of draining lymph nodes, with some of the cells also spreading deeper into the node (Fig. 2a). Localization of photoconverted cells in or near the subcapsular sinus strongly suggests that they entered the node via the lymphatics rather than the blood. Furthermore, when we examined neutrophil interactions with the lymphatic vessels in infected ears of Lysozyme M fluorescent reporter mice that allow visualization of neutrophils^9^, we detected neutrophils inside LYVE-1^+^ lymphatic vessels in inflamed ears (Fig. 2b). In addition, the first *in vivo* visualization of neutrophil migration inside the lymphatics was obtained (Fig. 2c and Supplementary Video 1).

We confirmed our observations of neutrophil migration in lymphatic vessels by transferring bone marrow from Lysozyme M fluorescent mice into Albino.BL/6 recipients (which are less susceptible to photodamage during imaging than black mice^24^) to obtain a fluorescently labelled population of ear neutrophils of >95% purity (Supplementary Fig. 3). Using this method, we found that neutrophils crawled inside the lymphatic vessels with a mean speed of 13 μm min^−1^ (Fig. 2d), frequently reversing direction of migration (Supplementary Videos 1 and 2). Although intralymphatic neutrophils were relatively infrequent (<1% of total neutrophils were found inside the lymphatics since the majority of neutrophils were clustered around foci of *S. aureus* infection), we also detected multiple neutrophils migrating in the same lymphatic vessel (Supplementary Video 2). Furthermore, neutrophils in lymphatic vessels were observed to carry fluorescently labelled *S. aureus* (Fig. 2e and Supplementary Video 2). Consistent with this, flow cytometric analysis demonstrated that neutrophils were the main cell type containing fluorescent bacteria in the ear as well as in draining lymph nodes. In contrast, few bacteria-containing cells could be detected in circulation and other organs (Fig. 2f and Supplementary Fig. 4). Together, these data indicated that *S. aureus*-carrying neutrophils were primarily directed to the draining lymph node via lymphatic vessels and only rarely underwent reverse transmigration to egress via blood vessels.

### Neutrophil migration to lymph nodes is independent of CCR7

**Figure 3:**
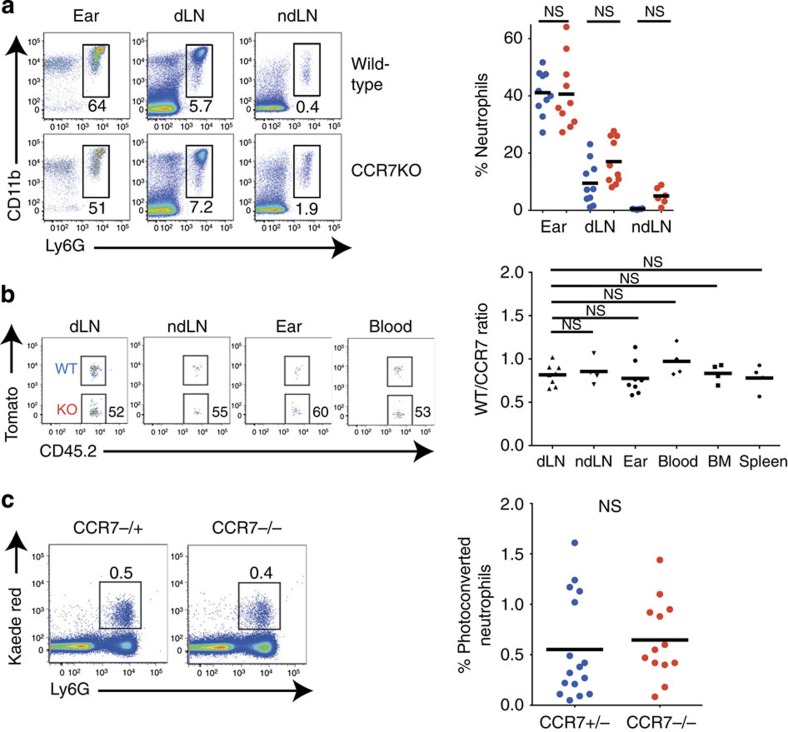
CCR7 is not required for neutrophil recruitment to skin and lymph nodes. (**a**) Percentages of neutrophils in ears and lymph nodes of wild-type and *Ccr7*^*−/−*^ mice 6–8 h after *S. aureus* injection. Representative fluorescence-activated cell sorting (FACS) plots gated on all live cells (left) and graphical summary of flow cytometric data from three independent experiments are shown (right). BM, bone marrow; dLN, draining lymph node; ndLN, non-draining lymph node. (**b**) Bone marrow from *Ccr7*^*−/−*^ mice (CD45.2 congenic variant) and wild-type mice (expressing CD45.2 and Tomato fluorescent protein in all cells) was intravenously transferred into wild-type (CD45.1 congenic variant) recipients. Mice were challenged with *S. aureus* for 8 h and transferred neutrophils in various organs were quantified by flow cytometry. The ratio between transferred wild-type and CCR7-deficient neutrophils is shown. (**c**) Kaede *Ccr7*^*−/−*^ and Kaede *Ccr7*^*+/−*^ mice were infected with *S. aureus*, and 4 h later, ears were photoconverted. A further 4 h later, mice were killed and photoconverted cells were detected by flow cytometry. Representative FACS plots (left) and graphical summary of flow cytometric data from four independent experiments are shown (right). Data were analysed using a one-way analysis of variance with a Bonferroni correction for multiple comparisons (**a**,**b**) or an unpaired student's *t*-test (**c**). NS, not significant.

Molecular events involved in neutrophil recruitment from the bloodstream to the site of inflammation have been well defined. However, the mechanisms responsible for neutrophil egress from inflamed tissue and their migration to draining lymph nodes are poorly understood. Since chemokine receptor CCR7 has been implicated in neutrophil entry into lymph nodes^25^, we examined inflamed lymph nodes in *Ccr7*^*−/−*^ mice. Unexpectedly, we did not observe any decrease in neutrophils in both draining and non-draining lymph nodes of *Ccr7*^*−/−*^ mice (Fig. 3a) suggesting that CCR7-deficient neutrophils readily entered lymph nodes. To determine whether CCR7-deficient neutrophils are at a competitive disadvantage with respect to lymph node homing, we intravenously co-transferred bone marrow from *Ccr7*^*−/−*^ mice (CD45.2 congenic variant) and wild-type Tomato transgenic mice (CD45.2 ROSA^mT/mG^ expressing Tomato fluorescent protein) into CD45.1 congenic recipients. The mice were then challenged with *S. aureus*. Flow cytometric analysis performed 6–8 h later failed to reveal any difference in wild-type and CCR7-deficient neutrophil recruitment to lymph nodes and other organs after challenge (Fig. 3b). Thus CCR7 expression was not required for neutrophil entry into lymph nodes.

Finally, we wanted to query specifically whether neutrophil migration from skin to lymph nodes is affected in the absence of CCR7. To take advantage of the photoconvertible system, CCR7-deficient mice were crossed to Kaede transgenic mice 26, which can be photoconverted from green to red using the same method as Kikume mice. These mice were challenged by ear injection of *S. aureus* and photoconverted as previously. A further 4 h later, mice were killed and photoconverted cells were detected in blood, draining lymph nodes and other immune organs using flow cytometry. CCR7-deficient and CCR7-heterozygous littermate control neutrophils migrated from skin to draining lymph nodes with similar efficiency (Fig. 3c). Thus CCR7 deficiency did not impair neutrophil migration from inflamed skin to draining lymph nodes.

### CD11b and CXCR4 modulate neutrophil migration from skin

**Figure 4:**
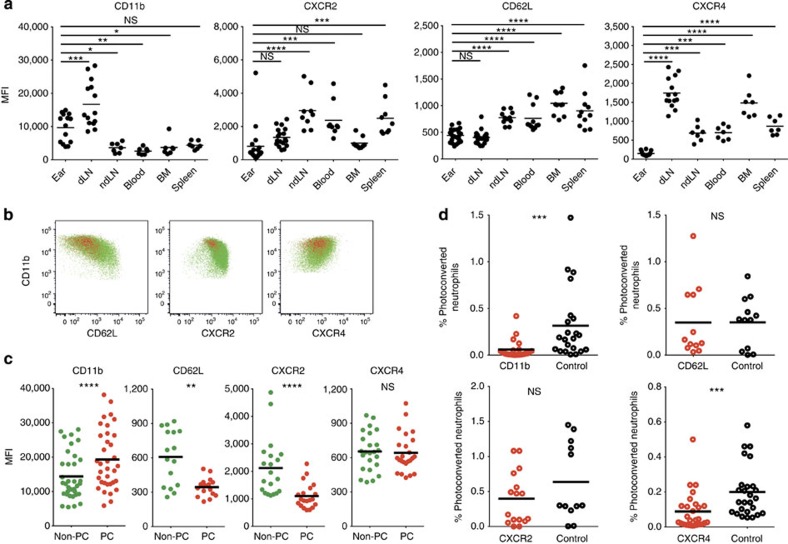
CD11b and CXCR4 mediate neutrophil migration from skin to lymph nodes. (**a**) Flow cytometric analysis of mean fluorescent intensities (MFIs) of the indicated molecules on neutrophils 8 h after an intradermal injection of *S. aureus*. BM, bone marrow; dLN, draining lymph node; ndLN, non-draining lymph node. (**b**) Representative fluorescence-activated cell sorting plots of CD11b, CD62L, CXCR2 and CXCR4 expression on photoconverted (red) and non-photoconverted (green) draining lymph node neutrophils 4h after photoconversion. (**c**) Flow cytometric analysis of CD11b, CD62L, CXCR2 and CXCR4 expression on photoconverted (PC, red circles) and non-photoconverted (non-PC, green circles) draining lymph node neutrophils 4 h after photoconversion and 8 h after *S. aureus* injection. Each point represents a single lymph node. Data from at least three independent experiments are shown. (**d**) CD11b, CD62L blocking antibodies or isotype controls or CXCR2 or CXCR4 inhibitors or vehicle controls were administered to the ears 4 h after *S. aureus* injection, ears were photoconverted immediately afterwards and percentages of photoconverted neutrophils out of all lymph node cells were determined 4 h later. Data were pooled from at least two independent experiments. Data were analysed using a one-way analysis of variance with a Bonferroni correction for multiple comparisons (**a**) or a paired student's *t*-test (**c**) or an unpaired student's *t*-test (**d**) *P≤0.05; **P≤0.01; ***P≤0.001; ****P≤0.0001; NS, not significant.

To identify alternative candidate molecules that may play a role in neutrophil migration to draining lymph nodes, we examined the phenotype of neutrophils in different organs after *S. aureus* challenge (Fig. 4a). We noted that draining lymph node neutrophils expressed elevated levels of the chemokine receptor CXCR4 as well as the integrin and complement receptor CD11b, while neutrophils in inflamed ears expressed lower levels of CD62L, CXCR2 and CXCR4 compared with other tissues (Fig. 4a).

We next examined the phenotype of lymph node neutrophils following *S. aureus* challenge and ear photoconversion. Photoconverted neutrophils that had recently entered the lymph node from inflamed skin could be distinguished on the basis of altered expression of CD11b, CD62L and CXCR2, while CXCR4 was expressed at similar levels in photoconverted and total neutrophils (Fig. 4b,c).

We tested the role of these molecules in migration from skin to draining lymph nodes. To differentiate between mechanisms of recruitment to inflamed tissue and subsequent egress from skin to lymph nodes, we first injected *S. aureus* into the ears of Kikume transgenic photoconvertible mice, allowed neutrophils to be recruited into the inflamed ear for 4 h and then administered blocking antibodies (anti-CD11b, -CD62L) or inhibitors of CXCR2 or CXCR4 intradermally into the ear. Inflamed ears were photoconverted immediately after and lymph nodes were analysed for the presence of photoconverted neutrophils after a further 4 h. Topical administration of anti-CD11b antibody to ears did not alter neutrophil uptake of *S. aureus* bioparticles (data not shown) but led to a 5-fold reduction in the numbers of Kikume red (recently-arrived) neutrophils in the draining lymph nodes (Fig. 4d) indicating that CD11b may play a role in neutrophil migration from skin to draining lymph nodes.

In contrast to anti-CD11b, localized administration of anti-CD62L failed to inhibit neutrophil migration from skin to draining lymph nodes, despite blocking recruitment from blood to inflamed ears when administered intravenously (Supplementary Table 1 and Supplementary Fig. 5). This indicates that the effect of anti-CD11b blockade on neutrophil migration is not due to IgG-mediated complement activation nor to generic effects of antibody binding on cell migration.

We confirmed a key role for CD11b in modulating neutrophil migration from skin to lymph nodes by transferring labelled bone marrow neutrophils from CD11b-deficient and wild-type mice directly into the ears of recipient mice that received *S. aureus* 4 h earlier. Flow cytometric analysis of ear and lymph node suspensions after a further 4 h showed a significant decrease in the ratio of CD11b-deficient neutrophils (relative to the co-transferred wild-type cells) in draining lymph nodes compared with ears (Supplementary Fig. 6), thus confirming that CD11b plays a role in neutrophil migration from inflamed skin to draining lymph nodes.

Localized CXCR2 blockade did not alter the migration of neutrophils from skin to the lymph nodes. However, administration of the CXCR4 inhibitor plerixafor/AMD3100 led to a significant reduction in the proportion of photoconverted neutrophils in draining lymph nodes. Notably, administration of pertussis toxin did not inhibit neutrophil migration from ears to draining lymph nodes (Supplementary Table 1) suggesting that CXCR4 may be modulating neutrophil migration from skin in a Gαi-independent manner.

We also used our photoconversion model to test several other molecules that may play a role in neutrophil migration via lymphatic vessels including ICAM-1 and LYVE-1 (expressed by lymphatic endothelial cells) and the integrin CD11a (involved in neutrophil migration to the site of inflammation, Supplementary Table 1). Targeting of these molecules had no effect on neutrophil migration from skin to draining lymph nodes, even though intravenous administration of ICAM-1 and CD11a prevented neutrophil recruitment to inflamed tissue (Supplementary Table 1) as was expected from published studies^3^. Thus, we have uncovered roles for both CD11b and CXCR4 in mediating neutrophil migration via lymphatic vessels from skin to draining lymph nodes.

### Neutrophils regulate early adaptive immune responses

**Figure 5:**
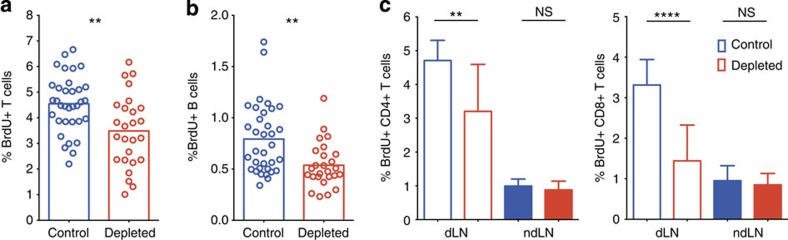
Neutrophils augment lymphocyte proliferation in draining lymph nodes. (**a**) C57BL/6 mice were treated with 50 μg of anti-Ly6G or isotype control antibody one day prior and at the time of intradermal injection of *S. aureus*, and proliferation was assessed by BrdU incorporation 72 h later. Percentage of CD3^+^BrdU^+^ cells is shown. (**b**) Percentage of B220^+^BrdU^+^ cells is shown after mice were treated as in **a**. (**c**) Percentage of BrdU^+^CD4^+^ and CD8^+^ T cells in draining and non-draining lymph nodes 3 days after injection of *S. aureus* into C57BL/6 mice treated with anti-Ly6G or isotype control antibody as in **a**. Means+s.d. are shown, three independent experiments with at least three mice per group were pooled. Data were analysed using an unpaired student's *t*-test (**a**,**b**) or a one-way analysis of variance with a Bonferroni correction for multiple comparisons (**c**) **P≤0.01; ****P≤0.0001; NS, not significant.

We next asked how neutrophils affect adaptive immune responses in draining lymph nodes. Neutrophils were depleted by two intraperitoneal injections of a neutrophil-specific anti-Ly6G antibody (50 μg 1 day prior as well as at the time of bacterial challenge) and lymphocyte proliferation was analysed by BrdU incorporation 3 days after *S. aureus* injection. Compared with animals that received control antibody, significantly fewer BrdU^+^ T and B cells were observed in neutrophil-depleted mice (Fig. 5a,b). This was true for both CD4^+^ and CD8^+^ T cells although the effect on CD8 T-cell proliferation was more pronounced (Fig. 5c). Thus neutrophils appeared to enhance early adaptive immune responses in draining lymph nodes.

This effect correlated with higher levels of MHC class II (Supplementary Fig. 7a) as well as the co-stimulatory molecules, CD80 and CD86 (Supplementary Fig. 7b) on draining lymph node neutrophils compared with neutrophils in other organs (with the exception of bone marrow). Furthermore, MHC class II expression on *S. aureus*-containing neutrophils was increased compared with neutrophils, which lacked bacteria and comparable to levels on Ly6G^−^CD11b^+^ cells (macrophages and dendritic cells, Supplementary Fig. 7c) suggesting a link between MHC class II expression and neutrophil phagocytosis and activation.

**Figure 6:**
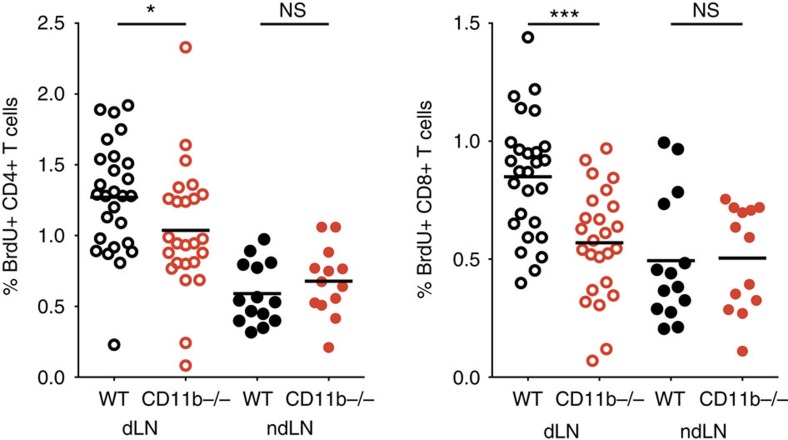
CD11b-dependent neutrophil migration modulates T-cell proliferation. CD11b-deficient and wild-type bone marrow neutrophils were incubated with *S. aureus* and injected into the ear pinna of neutrophil-depleted C57BL/6 mice. Proliferation was measured by BrdU incorporation 72 h later. Data were pooled from three independent experiments with at least four mice per group and analysed using a one-way analysis of variance with a Bonferroni correction for multiple comparisons. *P≤0.05, ***P≤0.001; NS, not significant.

Finally, we set out to show that specific inhibition of CD11b-mediated neutrophil migration from skin to draining lymph nodes could modulate early adaptive immune responses. To demonstrate this, we transferred *S. aureus*-pulsed CD11b-deficient or wild-type neutrophils directly into the ear pinna of mice previously depleted of neutrophils via a single injection of 50 μg of anti-Ly6G antibody. T-cell proliferation was assessed by BrdU incorporation 72 h later. We found that proliferation of both CD4^+^ and CD8^+^ T cells was significantly lower in response to CD11b-deficient neutrophils compared with wild-type neutrophils (Fig. 6). To ensure that this is not due to a functional defect in CD11b-deficient neutrophils, we also transferred wild-type *S. aureus*-pulsed neutrophils that had been pre-incubated with either a CD11b-blocking antibody or an isotype control. Both CD4^+^ and CD8^+^ T-cell proliferation was significantly reduced in mice that received neutrophils treated with anti-CD11b compared with control animals (Supplementary Fig. 8). Thus, we provide evidence that CD11b-dependent neutrophil migration from skin affects adaptive immune responses in draining lymph nodes. Since blocking CXCR4 also reduced neutrophil migration from skin to draining lymph nodes, we expect that inhibition of CXCR4 would lead to a similar decrease in T-cell proliferation in draining lymph nodes and would provide additional opportunities for modulating adaptive immune responses via regulation of lymphatic migration of neutrophils.

In conclusion, these data suggest that the rapid dissemination of neutrophils from the site of bacterial inflammation to secondary lymphoid organs may enhance lymphocyte responses, thereby providing an additional link between the innate and adaptive immune systems.

## Discussion

In addition to their well-known role in pathogen killing in inflamed tissues, recent evidence indicates that neutrophils may also affect adaptive immune responses in lymphoid organs^2^. We have established a photoconvertible transgenic system to track the fate of neutrophils recruited to sites of inflammation and used this system to create a comprehensive picture of the early events during anti-microbial immune responses. Neutrophils were rapidly recruited in response to both microbial and sterile challenges but after their arrival in inflamed skin, neutrophil phenotypes and fates diverged. Neutrophils responding to microbial lesions expressed higher levels of the activation and migration molecule CD11b, whereas neutrophils recruited to sterile lesions showed augmented expression of CXCR2 and CD62L. In bacterial infections, the signals generated in response to microbial stimuli are superimposed on top of the cell and tissue damage signals produced in sterile inflammation. Therefore, the altered phenotype of neutrophils responding to *S. aureus* compared with neutrophils in sterile lesions is likely due to a combined effect of tissue injury signals together with specific microbial stimuli that are absent in sterile inflammation.

The differences in molecules that guide neutrophil migration, in combination with the chemoattractant signals selectively generated in bacteria-containing lymph nodes led to distinct migration patterns. Specifically, neutrophils migrated from skin to draining lymph nodes only when bacteria were present at the primary lesion. As the main immune subset to leave the site of primary injury early in the response, neutrophils may be crucial not only for controlling pathogens that escape from skin but also for initial antigen transport from the site of invasion and the initiation of immune responses in draining lymph nodes. Conversely, this pathway may be exploited by pathogens since neutrophils can act as ‘Trojan horses' to aid the spread of infection^27^,^28^,^29^. Modulating neutrophil migration to the lymph nodes may, therefore, present an important avenue for therapeutic intervention in the treatment of inflammation and infection.

A recent study demonstrated a role for *S. aureus*-produced cytotoxin α-hemolysin in inhibiting neutrophil recruitment to inflamed skin^30^. We observed no significant difference in neutrophil recruitment to the ear in response to killed *S. aureus* compared with sterile injury suggesting that the level of α-hemolysin in killed *S. aureus* is not sufficient to inhibit neutrophil recruitment to the site of inflammation. However, it would be intriguing to determine whether α-hemolysin can modulate lymphatic migration of neutrophils between inflamed skin and draining lymph nodes.

With the aid of two-photon microscopy, we visualized neutrophil migration in lymphatic vessels of the skin and confirmed, using photoconversion, that neutrophils utilize lymphatics rather than reverse transmigration into blood vessels to migrate from skin to draining lymph nodes. This is consistent with observations obtained in *Mycobacterium bovis* BCG^8^ and *Toxoplasma gondii*^9^ infections. Furthermore, our photoconvertible system provided us with a unique opportunity to examine the mechanism of neutrophil migration from skin to draining lymph nodes. Interestingly, contrary to what was reported by Beauvillain *et al*.^25^ in Freund adjuvant model, we found both neutrophil egress from the skin and entry into the lymph nodes to be independent of CCR7. This is in contrast with CCR7-guided DC migration from skin to draining lymph nodes^31^ suggesting that a distinct mechanism is responsible for neutrophil egress from inflamed skin.

Chemokine receptors CXCR2 and CXCR4 have also been implicated in regulating neutrophil migration. CXCR2 mediates neutrophil migration along the vasculature^32^ and into tissues^33^ while CXCR4 regulates neutrophil release from the bone marrow^34^ and lungs^35^ and has also been implicated in immune complex-induced recruitment to lymph nodes^36^. Localized inhibition of these chemokine receptors showed that CXCR4 but not CXCR2 was involved in neutrophil migration from skin to draining lymph nodes. An earlier study demonstrated that CXCR4 engagement also promotes cutaneous DC migration to lymph nodes and that its ligand, CXCL12, is expressed in skin lymphatics^37^. Consistent with a prior report^19^, pertussis toxin did not inhibit neutrophil migration to lymph nodes. This suggests that CXCR4 is acting in a Gαi-independent manner as has been previously reported for this receptor^38^,^39^.

The mechanism of neutrophil egress from microbial lesions also appeared to involve CD11b since localized blockade of this molecule or complete removal of CD11b (in neutrophils from CD11b-deficient mice) inhibited neutrophil migration from skin to lymph nodes. CD11b is a highly versatile molecule that can mediate a wide range of biological functions^40^. It binds to ICAM-1 to mediate neutrophil migration out of the blood vessels to inflammatory sites^41^, forms part of complement receptor 3 and plays a role in neutrophil activation^40^. Other ligands of CD11b include factor X^42^, fibrinogen^43^ as well as junctional adhesion molecule JAM-C, which regulates neutrophil transendothelial migration^6^,^44^ and is expressed by blood and lymphatic endothelial cells^45^. Since ICAM-1 blockade failed to significantly diminish neutrophil migration from inflamed skin, CD11b may modulate lymphatic migration of neutrophils via its interactions with JAM-C or through its role in neutrophil activation via the complement pathway.

Interestingly, lymphatic migration of neutrophils was not inhibited by topical administration of anti-CD11a or anti-CD62L even though both of these antibodies effectively prevented neutrophil recruitment from blood to sites of microbial inflammation. Thus, lymphatic migration of neutrophils appears to be governed by mechanisms that are distinct from the well-characterized molecular processes responsible for neutrophil recruitment to sites of inflammation.

Following their arrival in the lymph node, photoconverted skin-egressing neutrophils disappeared within 36 h while photoconverted neutrophils in the skin persisted for several days. Neutrophil persistence in inflamed skin is consistent with an earlier observation of prolonged neutrophil survival in *S. aureus*-induced lesions^4^. The disappearance of neutrophils from lymph nodes appears to be due to apoptosis since we observed elevated levels of Annexin V staining in neutrophils located in draining lymph nodes. However, it is unclear what drives neutrophil apoptosis in lymph nodes and not the skin since both neutrophils that contain *S. aureus* and the ones that do not, show elevated levels of Annexin V staining on draining lymph nodes. Thus additional non-microbial factors present in inflamed skin but not in lymph nodes must contribute to neutrophil survival.

Despite their limited lifespan, rapid re-localization from the site of inflammation to draining lymph nodes gives neutrophils the opportunity to modulate early adaptive responses in lymph nodes before the arrival or activation of other innate immune cells. Consistent with this suggestion, we found that neutrophils enhanced B- and T-cell proliferation at the early time points following *S. aureus* administration. Furthermore, we demonstrate that T-cell proliferation in draining lymph nodes can be modulated by the specific inhibition of neutrophil migration from inflamed skin. This suggests that adaptive immune responses to microbes can be regulated by controlling neutrophil influx from the site of inflammation. Whether the effect on lymphocyte proliferation is mediated directly by lymph node neutrophils, which upregulate MHC class II and co-stimulatory molecules, shuttle antigen to lymph nodes and produce inflammatory mediators, or indirectly via other immune cells recruited or activated by neutrophils remains to be determined. Interestingly, a recent study has found that neutrophils had a suppressive effect on T-cell responses especially in distal lymph nodes^19^. However, this effect was observed 7 days after challenge coinciding with a second wave of neutrophil migration to the lymph nodes. Given the multitude of ways that neutrophils can affect immune responses, the precise effect of neutrophils on the adaptive immune response is likely to be highly dependent on the timing.

In conclusion, our study reveals the mechanisms of neutrophil-mediated communication between the site of initial injury and local lymph nodes where adaptive immune responses are initiated.

## Methods

### Mice

All mice used in this study were on the C57BL/6 background and were housed in specific-pathogen-free conditions. Both male and female animals of 8–12 weeks were used for these experiments. All animal experiments and procedures were approved by the Garvan Institute of Medical Research/St Vincent's Hospital Animal Ethics Committee. C57BL/6 mice were from Australian BioResources (Moss Vale, NSW). The ROSA-CAG-lox-stop-lox-KikGR knock-in and Kaede mice were a kind gift from Michio Tomura and were backcrossed and maintained on the C57BL/6 background. To generate whole-body Kikume transgenic mice ROSA-CAG-lox-stop-lox-KikGR mice were crossed to Rosa26 Cre (Jackson Laboratories). Albino.B6 or C57BL/6 mice with spontaneous mutations in the tyrosinase gene (B6(Cg)-*Tyr*^*c-2J*^/J) as well as CCR7^−/−^ and Lysozyme M Cre mice were obtained from Jackson Laboratories. Lysozyme M fluorescent reporter mice were generated by crossing Lysozyme M Cre mice to ROSA^mT/mG^ (Jackson Laboratories) mice. CD11b-deficient mice (B6.129S4-*Itgam*^*tm1Myd*^/J) were a kind gift from Dr Karlheinz Peter and were on the C57BL/6 background.

### Neutrophil isolation and bone marrow transfer

Bone marrow was harvested by flushing femurs and tibias of donor mice using 2% BCS+1 mM EDTA in RPMI. Erythrocytes were removed by lysing with 10 mM KHCO_3_, 0.1 mM EDTA and 166 mM NH_4_Cl and washing twice with RPMI in 2% BCS+1 mM EDTA. The resulting cell suspension was transferred intravenously into recipient mice in a volume of 200–300 μl at a ratio of three donors per one recipient. In some instances, neutrophils were purified from bone marrow using an EasySep Mouse Neutrophil Enrichment Kit from Stem Cell Technologies. To analyse the role of CD11b in neutrophil migration to lymph nodes, 2 × 10^5^ CD45.2 CD11b-deficient and Kaede wild-type neutrophils were transferred into the ears of CD45.1 congenic recipients, which were injected with *S. aureus* 4 h earlier. Mice were killed after a further 4 h and analysed by flow cytometry.

### Quantification of neutrophil migration from skin

Microbial inflammation was induced in the ears by injecting 2 × 10^7^ *S. aureus* bioparticles (Invitrogen) into the ear pinnae, non-microbial inflammation was induced by carefully scratching the ear skin with a sterile 29-gauge needle. Fluorescent bioparticles were generated by in-house labelling using Pacific Blue, Alexa Fluor 647 or Alexa Fluor 680 monoclonal antibody-labelling kits (Invitrogen).

Photoconversion was performed 4 h following the induction of inflammation. For skin photoconversion, ears were irradiated with violet light from a cold-light source fitted with a conversion filter (Zeiss) to minimize thermal damage and phototoxicity. Maximal increase in red fluorescence was achieved by irradiation for 20 min. Photoconverted Kikume red cells were detected by flow cytometry after a further 4 h.

### Blocking neutrophil migration from skin

Microbial inflammation was induced as above, and, 4 h later, ear pinnae of photoconvertible mice were injected using a 29-gauge needle with 5 μg of anti-CD11b, (clone M1/70), 10 μg of CD11a (clone M1/74), 10 μg of CD62L (clone Mel14), 10 μg of ICAM-1 (clone YN1/1.7.4), 10 μg LYVE-1( clone 223322, R&D systems) or 10 μg of isotype control antibodies, IgG2b (clone LTF-2, UCSF monoclonal antibody core) or IgG2a (clone 2A3, BioXcell) or SB225002 (Cayman Chemical) in dimethylsulphoxide diluted to 7 μg in 0.9% NaCl, 0.33% Tween 80 or 60 μg of plerixafor/AMD3100 in phosphate-buffered saline (PBS, Adooq Bioscience) or 500 ng of pertussis toxin (List Biological Laboratories) or PBS control, immediately before photoconversion. Draining and non-draining lymph nodes were collected 4 h after photoconversion, single-cell suspensions were created by mechanical disruption on a 100 μM filter and neutrophils were identified by Ly6G and CXCR2 expression.

### Blocking neutrophil recruitment from blood

The C57BL/6 mice were given either 170 μg of CD11a (clone M1/74), 20 μg CD11b (clone M1/70), 150 μg of CD62L (clone Mel14), 200 μg of ICAM-1 (clone YN1/1.7.4) or 200 μg of isotype control antibodies IgG2b (clone LTF-2 UCSF monoclonal antibody core) or IgG2a (clone 2A3), 500 ng of pertussis toxin or PBS control and then immunized with *S. aureus* and analysed 8 h later as previously described.

### Neutrophil regulation of T-cell proliferation

Twenty-four hours before induction of inflammation, mice were injected intraperitoneally with either 50 μg of rat-anti-mouse Ly6G (clone 1A8) or 50 μg of isotype control antibody (clone 2A3, BioXcell). Twenty-four hours later, 2 × 10^7^ *S. aureus* bioparticles were injected into the ear pinnae, mice were bled to confirm depletion and then injected intraperitoneally with either 50 μg of rat-anti-mouse Ly6G or isotype control antibody. To confirm depletion, blood was lysed and stained with purified Ly6G (clone 1A8), then goat-anti-rat-IgG Alexa-555 (Invitrogen), then CD11b-A647 in 2% normal rat serum. Forty-eight and 60 h later, mice were injected with 100 μl BrdU in PBS (0.8 mg ml^−1^, Sigma-Aldrich) intraperitoneally. Seventy-two hours after *S. aureus* administration, mice were killed.

To test the role of CD11b-mediated neutrophil migration in T-cell proliferation, neutrophils were purified from the bone marrow of CD11b-deficient or wild-type mice and resuspended in DMEM+2% FCS+2 mM EDTA at a concentration of 2 × 10^7^ cells ml^−1^. A total 4 × 10^8^ *S. aureus* bioparticles per ml were added to neutrophils and this mixture was incubated at 37 degrees for 15 min, cells were washed and 5 × 10^5^ CD11b-deficient and wild-type neutrophils were transferred into the ear pinna of C57BL/6 mice that were depleted of neutrophils by a single injection of anti-Ly6G (50 μg) 24 h earlier. Proliferation was measured by BrdU incorporation 72 h later as described. Alternatively, wild-type neutrophils were isolated from bone marrow resuspended at 2 × 10^7^ cells per ml and then incubated with 4 × 10^8^ *S. aureus* bioparticles per ml at 37 degrees for 15 min, neutrophils were washed and then incubated with 1 μg of anti-CD11b or isotype control for 15 min on ice, 5 × 10^5^ neutrophils were transferred into the ear pinnae of C57BL/6 mice made neutropenic by a single injection of anti-Ly6G (50 μg). Proliferation was measured by BrdU incorporation 72 h later as previously described.

### Two-photon intravital microscopy

Two-photon imaging was performed using an upright Zeiss 7MP two-photon microscope (Carl Zeiss) with a W Plan-Apochromat 20 × /1.0 DIC (ultraviolet) Visible-IR water immersion objective. Four external NDDs were used to detect blue (SP 485), green (BP 500–550), red (BP 565–610) and far red (BP 640–710). High repetition rate femtosecond-pulsed excitation was provided by a Chameleon Vision II Ti:Sa laser (Coherent Scientific) with 690–1,064 nm tuning range. In some experiments, ∼8 × 10^6^ fluorescent Lysozyme M bone marrow cells were injected intravenously 16h before *S. aureus* challenge into Albino.B6 recipient mice to generate ∼95% pure population of fluorescent neutrophils in the ear. *In vivo* labelling of lymphatic endothelial cells was achieved by injecting 1–5 μg of anti-LYVE-1-Alexa Fluor 647 into the ear 30 min before imaging.

We acquired 3 μm z-steps at 512 × 512 pixels and resolution 0.83 μm per pixel at a frame rate of 10 fps and dwell time of 1.27 μs per pixel using bidirectional scanning.

Intravital two-photon microscopy was based on a previously described method^23^. Anesthesia was induced with 100 mg kg^−1^ ketamine/5 mg kg^−1^ xylazine and maintained with 1–2% isoflurane supplemented with 100% oxygen at a flow rate of 500 ml min^−1^ via a nose cone. Anaesthetized mice were kept warm using a customized heated SmartStage (Biotherm). The ear was immobilized on a base of thermal conductive T-putty (Thermagon Inc.) using Vetbond tissue adhesive (3 M).

### Image processing and data analysis

Raw image files were processed using Imaris (Bitplane) software. A Gaussian filter was applied to reduce background noise. Tracking was performed using Imaris spot detection function to locate the centroid of cells. Motility parameters such as cell displacement (or track length calculated as the total length of displacements within the track) and track speed (calculated by dividing track length by time) were obtained using Imaris Statistics function. All modelling and statistical analysis was performed in GraphPad Prism. Means between two normally distributed groups were compared using a Student's *t*-test.

### Antibodies and flow cytometry

Single-cell suspensions were created by mechanical disruption and passed through a 100 μM filter, erythrocytes were removed by lysing with 10 mM KHCO_3_, 0.1 mM EDTA and 166 mM NH_4_Cl solution. If required, ears and lymph nodes were digested with 5 mg of Collagenase D (Roche) and five units of RQ1 DNase (Promega) in DMEM. Cells (2 × 10^6^) were stained with antibodies in 100 μl of 1 × PBS+0.2% BSA and 0.1% NaN_3_+2 mM EDTA. Ly6G (clone 1A8), CD3-biotin (clone 145-2C11), CD11b (clone M1/70), CD4 (clone GSK1.5), CD8 (clone YTS169.4) and F4/80 biotin (clone BM8) were obtained from UCSF monoclonal antibody core, labelled in-house with either Pacific Blue or Alexa Fluor 647 using antibody-labelling kits (Invitrogen) and used at 2.5–5 μg ml^−1^. Anti-BrdU-FITC (clone B44, 1.3 μg ml^−1^) was purchased from Becton Dickinson. Streptavidin PE (1 μg ml^−1^), CD80-Biotin (clone 16-10A1, 1 μg ml^−1^), CD86-Biotin (clone GL1,1 μg ml^−1^), MHC class-II-PE (clone AF6–120.1, 0.4 μg ml^−1^), AnnexinV-APC (5 μl per sample), Annexin V-PE (5 μl per sample) and CD11c-PE-Cy7 (clone HL3, 0.66 μg ml^−1^) were purchased from BD Pharmingen. CD11b-BV711 (clone M1/70, 0.4 μg ml^−1^) was obtained from BD Horizon. β_2_-Microglobulin-FITC (clone 9S19.8, 0.02 μg ml^−1^) was bought from Santa Cruz Biotechnology. CD11b-Pacific Blue (clone M1/70, 0.5 μg ml^−1^), CD11b-Alexa Fluor 647 (clone M1/70, 0.66 μg ml^−1^), Fc block (clone 93, 1.3 μg ml^−1^) and streptavidin PE-Cy7 (0.2 μg ml^−1^) were purchased from eBioscience. Purified CXCR2 (clone 242216, 2.5 μg ml^−1^ R&D systems) was labelled in house. Goat-anti-Rat IgG-Alexa Fluor 555 (4 μg ml^−1^) was purchased from Invitrogen. Mouse serum for blocking was obtained from C57BL/6 mice or purchased from Jackson ImmunoResearch. CD3 PE-Cy7 (clone 17A2, 1 μg ml^−1^) was bought from Biolegend. Samples were stained with DAPI (diluted 1/100,000), to exclude dead cells and data were acquired on an LSRII (BD Biosciences). Data were analysed using FlowJo software (Tree Star Inc).

### Statistical analysis

The statistical significance of experimental results was determined using either unpaired or paired two-tailed student's *t*-test for comparisons of two groups. One-way or two-way analysis of variance with Bonferroni correction for comparisons of multiple groups was used to compare three or more groups. All the tests were performed using GraphPadPrism 6.0b (GraphPad Software).

## Additional information

**How to cite this article:** Hampton, H.R. *et al*. Microbe-dependent lymphatic migration of neutrophils modulates lymphocyte proliferation in lymph nodes. *Nat. Commun.* 6:7139 doi: 10.1038/ncomms8139 (2015).

## Supplementary Material

Supplementary Information Supplementary Figures 1-8 and Supplementary Table 1

Supplementary Movie 1 Neutrophils migrate inside lymphatic vessels in the skin. A lysozyme M GFP+ neutrophil (green) migrates with frequent stopping and turning inside LYVE-1+ lymphatic vessels (white). Red track indicates neutrophil path. Projection of a time-lapse series imaged in the ear skin of a Lysozyme M GFP mouse 4 hours after S. aureus injection. Dimensions: 120x116x22 μm Elapsed time is shown as days:hours:min:seconds. Tick marks are 10 μm apart.

Supplementary Movie 2 Neutrophils carry S. aureus inside lymphatic vessels. Transferred lysozyme M GFP+ neutrophils (green) migrate inside LYVE-1+ lymphatic vessels (white). One of the neutrophils carries S. aureus particles (blue). Collagen/Second harmonic (blue). Red tracks indicate neutrophil paths. Projection of a time-lapse series imaged in the ear skin of an albino.BL6 mouse (which has received lysozyme M GFP+ bone marrow 24 hours earlier) 4 hours after S. aureus injection. Dimensions: 137x137x14 μm. Elapsed time is shown as days:hours:min:seconds. Tick marks are 10 μm apart.
