# Supplementary material for: Microbe-dependent lymphatic migration of neutrophils modulates lymphocyte proliferation in lymph nodes
Source: Nat Commun. 2015 May 14;6:7139. doi: 10.1038/ncomms8139 (PMC4479041; doi:10.1038/ncomms8139)
Supplement: Supplementary Information — Supplementary Figures 1-8 and Supplementary Table 1 [file ncomms8139-s1.pdf]

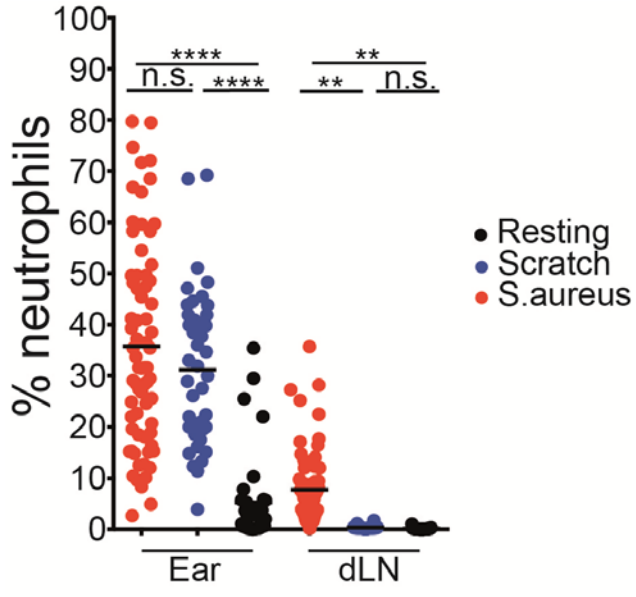

Supplementary Figure 1. Neutrophil recruitment to inflamed skin and draining lymph nodes during microbial and sterile inflammation. Flow cytometric analysis of mouse ears and draining lymph nodes (dLN) 8 hours after ear pinnae were either injected with killed *S. aureus* •, scratched • with a sterile needle or resting •. % of cells that are neutrophils ($Ly6G^{+}CD11b^{+}$) out of all live cells is shown. Data were pooled from more than 6 independent experiments with at least 2 mice per group. Recruitment was analysed by two-way ANOVA with a Bonferroni correction for multiple comparisons. ** $P \leq 0.01$; **** $P \leq 0.0001$; n.s. not significant.

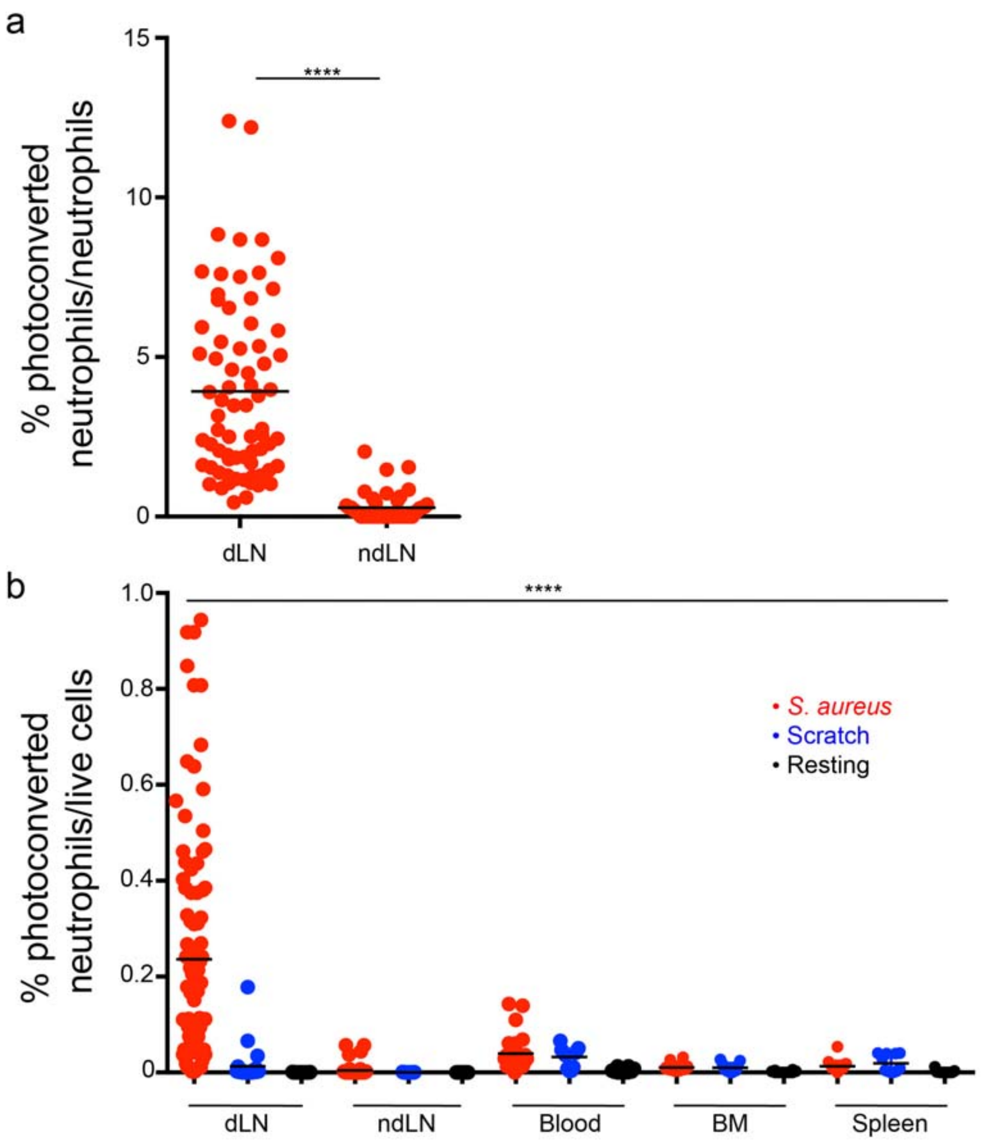

Supplementary Figure 2. Neutrophils egress microbial but not sterile lesions and migrate to draining lymph nodes. (a) Percentage of photoconverted neutrophils out of all neutrophils in the draining lymph nodes – dLN and non-draining lymph nodes – ndLN, of mice that received *S. aureus* and ears were photoconverted as in Fig. 1c. (b) Percentage of photoconverted neutrophils out of all live cells in the specified tissues, bone marrow – BM, after *S. aureus*–injected •, scratched • or resting • ears were photoconverted as in Fig.1c. Data were pooled from six independent experiments and analysed using an unpaired student's t-test (a) or a one-way ANOVA with a

Bonferroni correction for multiple comparisons (b). **** $P \leq 0.0001$. Individual *p*-values are not show in (b) since *p*-values were ≤ 0.0001 when *S. aureus* draining lymph nodes were compared to all other groups on the graph.

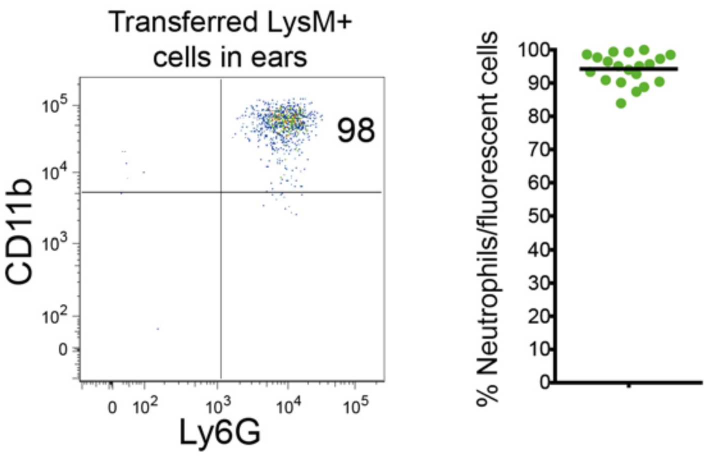

Supplementary Figure 3. Purity of transferred fluorescent neutrophils in mouse ears after intravital imaging. ~$8x10^7$ fluorescent Lysosyme M bone marrow cells were injected i.v. 16 hours prior to *S. aureus* challenge and subsequent imaging. Ear tissue was analysed after imaging by flow cytometry and the percentage of neutrophils out of all fluorescent cells was determined. Each circle represents an imaged ear.

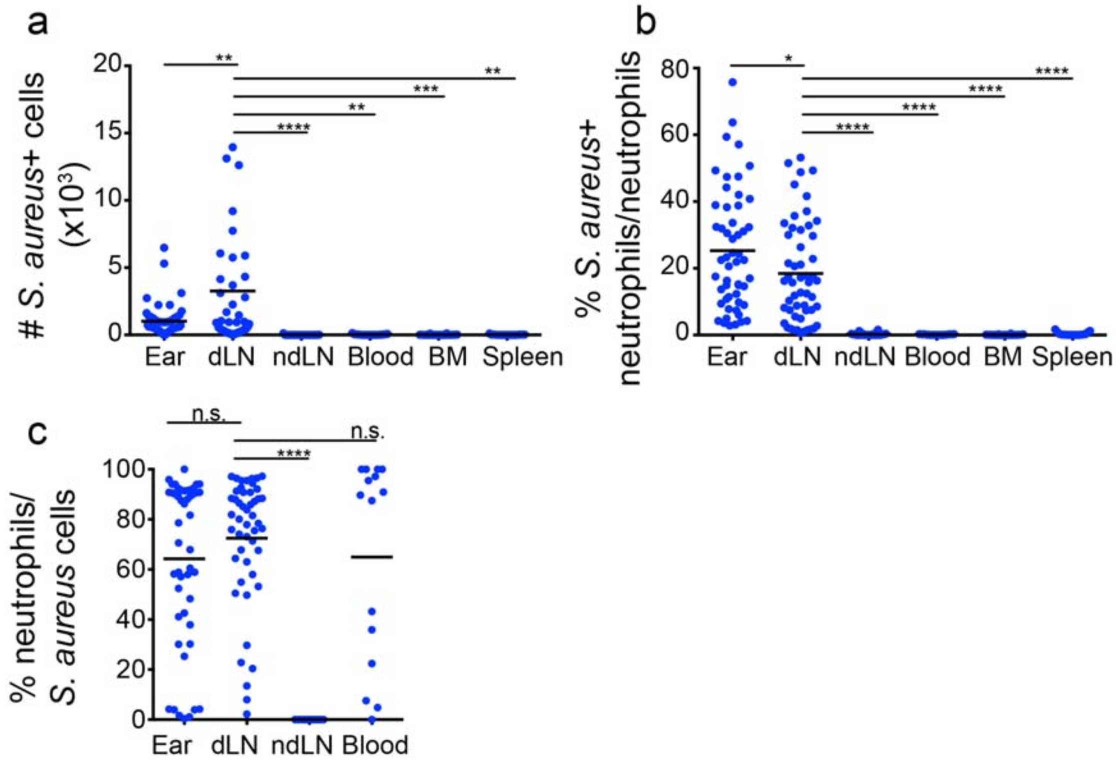

Supplementary Figure 4. Neutrophils in the skin and draining lymph node contain *S. aureus*. C57BL/6 mice were injected with fluorescently labeled *S. aureus* and phagocytosis was assessed 8 hours later. (a) Number of *S. aureus*+ cells in the indicated organs: draining lymph nodes – dLN; non-draining lymph nodes – ndLN; bone marrow – BM. (b) % of neutrophils that are *S. aureus*+ out of all neutrophils in the specified tissues. (c) Percentage of *S. aureus*+ neutrophils out of *S. aureus*+ cells in the indicated organs. Data was pooled from 7 experiments with least two mice and was analysed using a one-way ANOVA with a Bonferroni correction for multiple comparisons. *$P \leq 0.05$; **$P \leq 0.001$; ***$P \leq 0.001$; ****$P \leq 0.0001$; n.s. not significant.

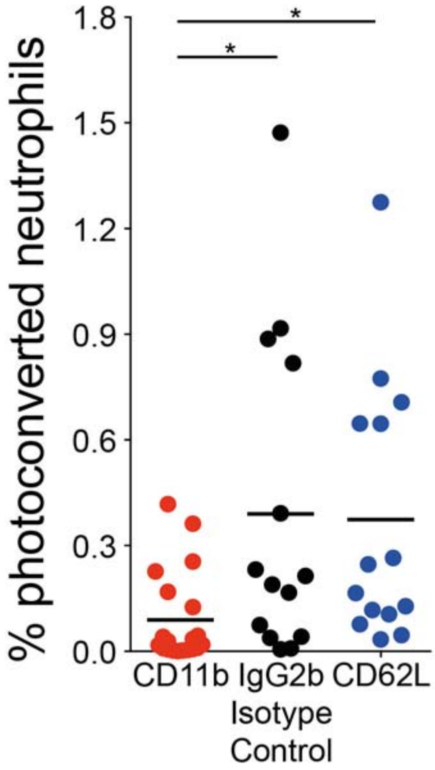

Supplementary Figure 5. Anti-CD11b but not anti-CD62L inhibits neutrophil migration from the skin to draining lymph nodes. *S. aureus* was introduced into the ears of Kikume mice, four hours later anti-CD11b •, IgG2b Isotype control • or anti-CD62L • was injected into the ears, which were then photoconverted. Neutrophil egress to the draining lymph node was assessed four hours later by flow cytometry. Graph shows the % of all cells that are red neutrophils out of all lymph node cells. Data is pooled from at least two independent experiments. Data was analysed by one-way ANOVA with a Bonferroni correction for multiple comparisons. * $P \leq 0.05$.

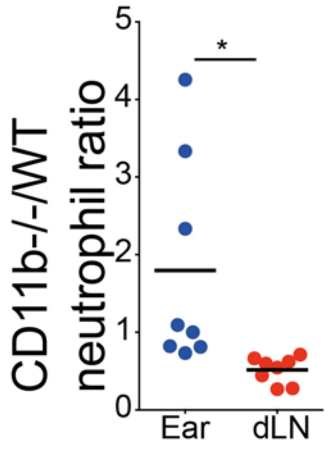

Supplementary Figure 6. CD11b deficiency inhibits neutrophil migration from the skin to the draining lymph node. *S. aureus* was injected into the ear pinnae of CD45.1 (congenic) mice. Four hours later bone marrow neutrophils isolated from CD45.2+ Kaede and CD45.2+ CD11b-deficient mice were transferred into the ear of CD45.1 congenic mice recipients. Four hours later donor neutrophils were detected in draining lymph nodes by flow cytometry. The ratio of CD11b to WT neutrophils in the indicated tissues from two independent experiments is shown. Data was analysed using an unpaired student's t-test. $*P \leq 0.05$

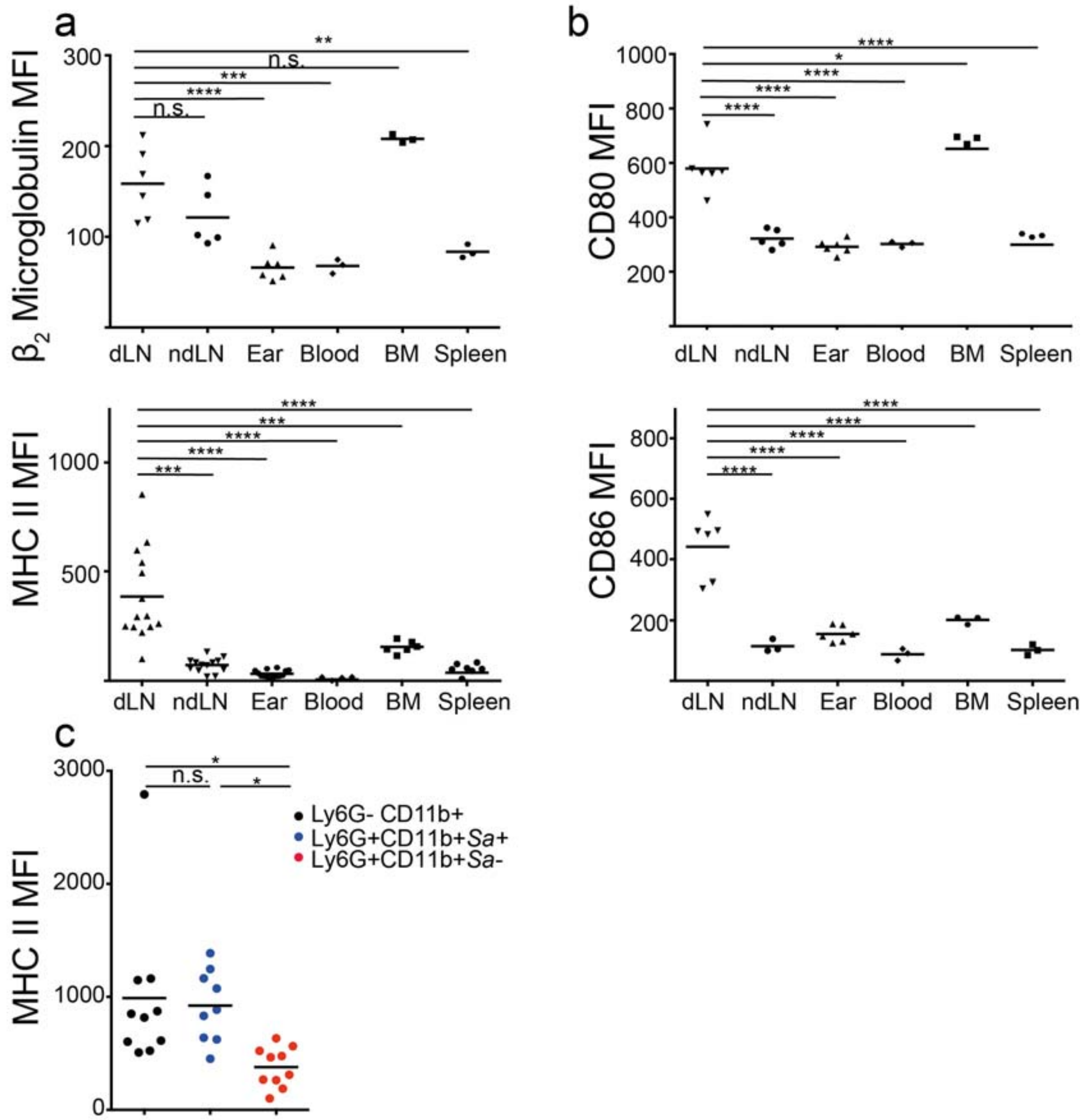

Supplementary Figure 7. Lymph nodes neutrophils express molecules associated with antigen presentation following an intradermal injection of *S. aureus*. (a) Flow cytometric analyses of mean fluorescent intensities (MFIs) of $ß_2$ microglobulin, and MHC class II or (b) CD80 and CD86 expression on neutrophils in different organs 8 hours after ear *S. aureus* infection; draining lymph node – dLN; non-draining lymph node – ndLN; bone marrow – BM. (c) MHC class II levels on *S. aureus*$^+$ • and *S. aureus*$^-$ • neutrophils as well as on Ly6G$^-$CD11b$^+$ cells • in the draining lymph nodes 8 hours after injection of fluorescent *S. aureus*. $ß_2$ microglobulin, CD80, CD86 MFIs are from a single experiment with three mice.

MHC-II MFI and (c) are from at least two experiments with 3 mice per group. Data was analysed using a one-way ANOVA with a Bonferroni correction for multiple comparisons. *$P \leq 0.05$; **$P \leq 0.01$; *** $P \leq 0.0001$, **** $P \leq 0.0001$; n.s. not significant.

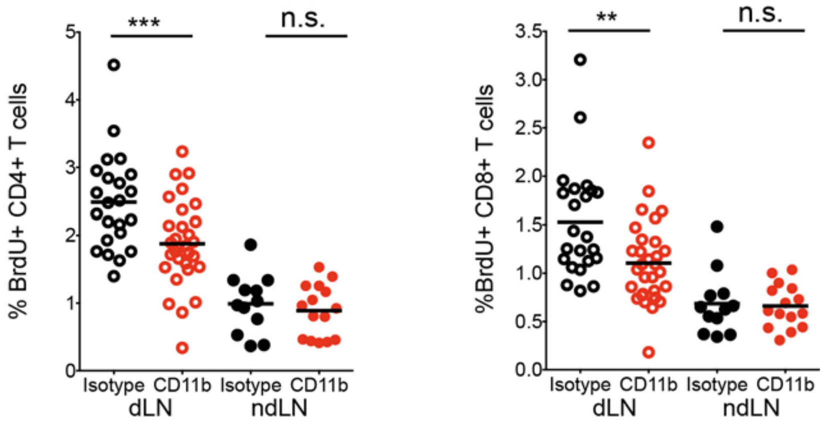

Supplementary Figure 8. Blockade of CD11b-mediated neutrophil migration from the skin to draining lymph nodes reduces T cell proliferation. Bone marrow neutrophils were pulsed with *S. aureus*, incubated with either anti-CD11b or an isotype control antibody and injected into the ear pinna of neutrophil depleted C57BL/6 mice. Proliferation was measured by BrdU incorporation 72 hours later in the draining-dLN and non-draining lymph node-ndLN. Data was pooled from three independent experiments normalised to dLN isotype control and analysed by a one-way ANOVA with a Bonferroni correction for multiple comparisons. ***P*≤ 0.01, ****P*≤ 0.001, n.s. not significant.

Supplementary Table 1. Molecular mechanisms of neutrophil migration to lymph nodes.

Molecule	Inhibit recruitment to inflamed skin and draining lymph nodes when administered intravenously?	Inhibit migration from inflamed skin to draining lymph nodes when administered to inflamed skin?
**CD11a**	Yes	No
**CD11b**	Yes	Yes
**CD62L**	Yes	No
**CXCR2**	No	No
**CXCR4**	Not tested	Yes
**ICAM-1**	Yes	No
**LYVE-1**	Not tested	No
**Pertussis toxin**	No	No

To analyse neutrophil egress from skin, Kikume mice were administered inhibitors or blocking antibodies into the ears 4 h after an intradermal injection of *S. aureus,* ears were then immediately photoconverted. Neutrophil migration from the skin to the draining lymph node was determined by flow cytometry 4 hours later. CD11b, CXCR4 and ICAM-1 – four independent experiments, CD11a, CD62L, CXCR2 and pertussis toxin - two independent experiments. Draining lymph nodes from treated and control mice were compared using an unpaired student's t-test or a Mann-Whitney U-test. To test the role of specific molecules in neutrophil recruitment from blood, C57BL/6 mice were administered inhibitors or blocking antibodies intravenously at the same time as *S. aureus* was injected into the ear pinnae and recruitment to lymph nodes was determined 8 h later by flow cytometry. Ears and draining lymph nodes from antibody-treated and isotype control treated mice were compared using a two-way ANOVA with a Bonferroni correction for multiple

comparisons. P-values ≤ 0.05 were considered statistically significant.
